# How to Approach Patients with Acute Chest Pain

**DOI:** 10.31083/j.rcm2508302

**Published:** 2024-08-22

**Authors:** Kenji Inoue, Tohru Minamino

**Affiliations:** ^1^Department of Cardiovascular Biology and Medicine, Juntendo University Nerima Hospital, 177-0033 Tokyo, Japan; ^2^Department of Cardiovascular Biology and Medicine, Juntendo University School of Medicine, 113-8421 Tokyo, Japan

**Keywords:** chest pain, emergency department, high sensitive cardiac troponin, 0-hour/1-hour algorithm

## Abstract

Acute coronary syndrome (ACS) is associated with high mortality rates. Although the goal was to achieve a missed diagnosis rate of <1%, the actual data showed a rate of >2%. Chest pain diagnosis has remained unchanged over the years and is based on medical interviews and electrocardiograms (ECG), with biomarkers playing complementary roles. We aimed to summarize the key points of medical interviews, ECG clinics, use of biomarkers, and clinical scores, identify problems, and provide directions for future research. Medical interviews should focus on the character and location of chest pain (is it accompanied by radiating pain?) and the duration, induction, and ameliorating factors. An ECG should be recorded within 10 minutes of the presentation. The serial performance of an ECG is recommended for emergency department (ED) evaluation of suspected ACS. Characteristic ECG traces, such as Wellens syndrome and De Winter T-waves, should be understood. Therefore, troponin levels in all patients with suspected ischemic heart disease should be examined using a highly sensitive assay system. Depending on the ED facility, the patient should be risk stratified by serial measurements of cardiac troponin levels (re-testing at one hour would be preferred) to determine the appropriate time to perform an invasive strategy for a definitive diagnosis. The diagnostics should be based on Bayes’ theorem; however, care should be taken to avoid the influence of heuristic bias.

## 1. Introduction

Chest pain is one of the most frequent causes of visits to emergency departments (ED) and general outpatient clinics. Differential diagnosis requires prompt and accurate evaluation of (1) ischemic heart disease, (2) other cardiovascular diseases (aortic dissection and pulmonary embolism), and (3) non-cardiovascular and pulmonary diseases [1, 2, 3]. Among these, acute coronary syndrome (ACS) with unclear electrocardiogram (ECG) changes accounts for approximately 10% of cases. The physician in charge decides whether a patient needs to be admitted to the hospital for diagnosis. However, differences in the accurate diagnosis rate depend not only on the physician’s skill but also on their personality, experience with medical errors, local customs, and patient background. Waxman *et al*. [4] investigated all Medicare claims in California and showed the number of patients discharged directly from the ED without a diagnosis of acute myocardial infarction (AMI); their analysis from 2006 to 2014 consistently found a missed diagnosis rate of >2%. However, excessive hospitalization and non-invasive examinations result in higher healthcare costs [5, 6, 7]. Hospital beds are a precious resource, as evidenced by the severe acute respiratory syndrome coronavirus 19 pandemic. Natsui *et al*. [8] analyzed a database of nearly 40,000 patients obtained over two years from 15 EDs in the United States. The admission rate for follow-up was 14%; however, there was a wide variation between physicians who tended to admit patients and those who did not (8.8%–93.7%). However, it did not affect the frequency of death or AMI within 30 days. Recently, the 2021 American Heart Association/American College of Cardiology/American Society of Echocardiography/American College of Chest Physicians/Society for Academic Emergency Medicine/Society of Cardiovascular Computed Tomography/Society for Cardiovascular Magnetic Resonance guidelines for the evaluation and diagnosis of acute chest pain were revised for the first time in nine years, to reduce the ACS miss rate to <1%, which was achieved by a combination of (1) medical interviews, (2) ECGs, and (3) biomarkers [9].

## 2. An Analogy is Drawn from the Medical Interviews

Harrison’s internal medicine study found that (1) gastrointestinal problems (e.g., reflux esophagitis) (42%), (2) ischemic heart disease (31%), and (3) musculoskeletal system disorders (28%) were the most common causes of chest pain. The characteristics of chest pain are essential in formulating an initial assessment. However, not all patients may describe chest pain; instead, they may claim chest discomfort or dyspnea. Surprisingly, the only highly specific finding with a high likelihood ratio suggestive of ACS was radiating pain to the (1) right shoulder or upper extremity and (2) bilateral shoulders or upper extremities being the most common causes. Other symptoms were not specific to ACS [10]. In contrast, pain triggered by palpitations, sharp pain, pain that changes with posture, and pain aggravated by inhalation are complaints that can rule out ACS (negative likelihood ratio <0.2). However, patients commonly describe chest pain as dyspnea or chest discomfort, rather than chest pain. A patient background approach that includes coronary risk factors may be effective in such cases. Particular attention should be paid to the presence or absence of hypertension, history of smoking, and statin use.

## 3. Using Analogies from Electrocardiographic Changes

It is important to record an ECG within 10 minutes of arrival at the hospital and to repeat the recording (within 30 to 60 minutes).

The strict definition of ST-segment elevation is the classical rule: ‘ST-segment elevation of more than 1 mm in two or more inductions’. In this case, ‘two or more inductions’ means anatomically adjacent inductions. The left precordial leads or II/III/aVF in limb leads are objective, and these two patterns cover almost 80% of ST-segment elevation myocardial infarction (STEMI). The definition of height is ‘more than 1 mm’, however, in the US, an ironclad rule of ‘more than 2 mm’ is imposed only on V2 lead and V3 lead, where false positives are more common; on V2 lead and V3 lead, the difference between men and women is more maniacal, with 1.5 mm (female) and 2.0 mm (male), or sometimes 2.5 mm for patients under 40 years old. The definition is very detailed, but it is not all about sensitivity. These detailed definitions are all rules to increase specificity without reducing sensitivity to prevent false positives [11].

**Fig. 1.**
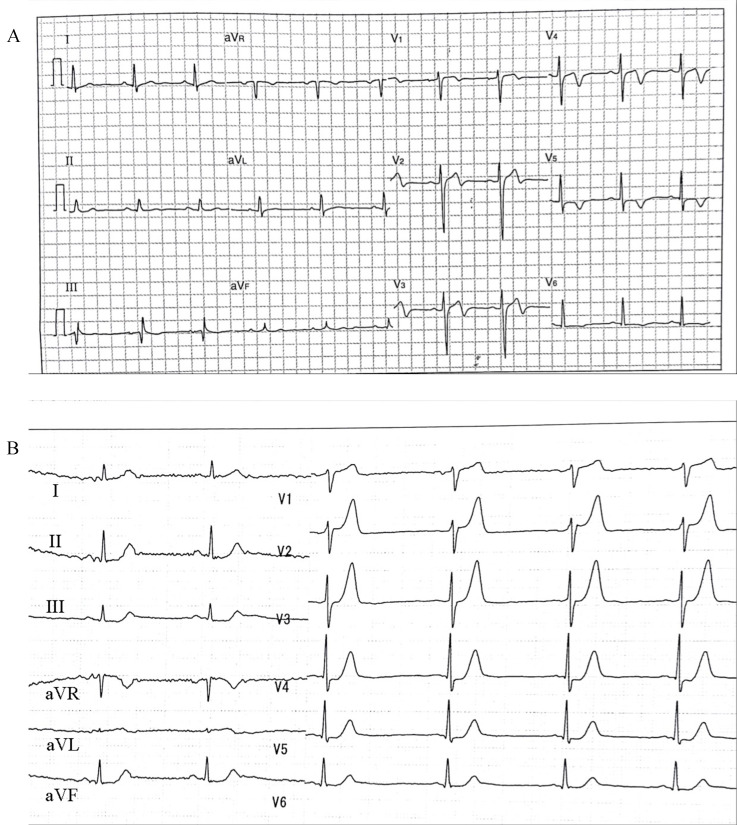
**The typical electrocardiogram (ECG) that should be considers for urgent coronary angiography**. (A) Wellens syndrome. (B) De Winter T waves.

Typical ST-segment elevation with reciprocal change (mirror phenomenon) is observed in only approximately 5% of cases [12, 13, 14]. In reality, assessing ischemic heart disease based on ST–T changes is challenging [15]. In this context, this review introduces some characteristic ECG findings identified by cardiologists. The first is Wellens’ syndrome, in which characteristic ECG changes are easily observed in leads V2–3 [16]. A slight upward shift and a negative wave at the end of the T wave characterized the T wave. The diagnosis was highly significant as the culprit’s vessel was the left anterior descending artery. Prescribing aspirin and immediately transferring the patient to a facility with catheterization capabilities should be considered (Fig. 1A). The second is De Winter T-waves, in which the ST portion falls by >1 mm, and sharp T-waves are observed under thoracic guidance. The culprit vessel was in the left anterior descending artery (Fig. 1B) [17].

## 4. The Analogy is Drawn from the Biomarkers

Diagnostic criteria for AMI have shifted from an epidemiological/pathological perspective to a biochemical (biomarker) perspective since the 20th century. The most important reason for this was the emergence of high-sensitivity troponin assays [18, 19, 20]. In 2009, data comparing the diagnostic efficiency of various high-sensitivity troponin assay systems for AMI were reported, and the Roche and Abbott assay systems showed an outstanding area under the curve (AUC) of around 0.95 for both I and T (AUC >0.8 is considered a high diagnostic performance) [21]. However, this AUC is low in clinical practice [22]. Troponin is known to be elevated when the oxygen demand is unbalanced, such as in tachyarrhythmia and anemia, cardiac diseases such as heart failure and cardiomyopathy, renal failure, and pulmonary embolism. ACS cannot be determined from a single troponin measurement.

## 5. High-sensitive Troponin Measurement System

Subsequently, various manufacturers developed troponin assays. They have been developing high-sensitivity assay systems according to their own unique standards. Therefore, the International Federation of Clinical Chemistry defines the following criteria as standards for high-sensitivity assay systems: [23]

(1) The 99th percentile should be determined in a healthy population.

(2) The 99th percentile for high-sensitivity cardiac troponin (hs-cTn) assays should be measured with an analytical imprecision of ≤10% (%CV; coefficient of variation).

(3) High-sensitivity assays should measure cardiac troponin above the detection limit in ≥50% of healthy participants.

## 6. Definition of AMI

The Fourth Universal Definition of Myocardial Infarction consensus document classifies AMI into types 1 to 5 and differentiates AMI from myocardial injury by providing the diagnostic term [18].

### 6.1 Myocardial Injury 

An abnormal troponin level (99th percentile or higher) was detected at least once among multiple measurements. A rise and/or fall in the cardiac troponin level was considered acute injury.

### 6.2 Myocardial Infarction

AMI is a myocardial injury characterized by one of the following features: (1) ischemic symptoms, (2) new electrocardiographic changes, (3) abnormal Q waves, (4) new regional wall motion abnormalities observed on echocardiography, or (5) thrombus identification on angiography.

The most frequent subtypes are type 1 myocardial infarction (MI), caused by acute plaque disruption or erosion; type 2 MI, caused by a myocardial imbalance in oxygen supply or demand; and myocardial injury, defined by an elevated concentration of the cardiac biomarker troponin in the absence of acute myocardial ischemia. In the ED, accurately discriminating between patients with type 1 MI, type 2 MI, and myocardial injury is challenging. This is probably due to the ischemic symptoms described above and the fact that identifying new ECG changes takes work in actual clinical practice. In other words, when a patient with anemia or tachyarrhythmia expresses chest tightness, the physician decides whether to consider ischemic symptoms. Similarly, in the case of renal impairment, no clear threshold has been established at which the level of numerical deterioration of troponin becomes a false-positive [24, 25, 26]. Therefore, it is difficult to determine whether abnormal troponin levels indicate ACS.

## 7. Risk Scores

Among those without significant ECG changes and abnormal troponin values, only 1% to 4% of these patients have ACS. Risk scores are helpful given the relatively low yield of the classical approach for possible ACS. Of these, the History, Electrocardiogram, Age, Risk factors and Troponin (HEART) score and the Emergency Department Assessment of Chest Pain Score (EDACS) are recommended in the guidelines and are widely used. The HEART score is characterized by incorporating the impression of the patient’s condition and ECG, which physicians rely on, and guidelines emphasize the importance of the score.

However, the HEART score incorporates the judgment of the attending physician into the evaluation, which may introduce bias. On the other hand, the EDACS is somewhat age-weighted. For example, a 50-year-old man with a history of smoking who presents with chest pain, sweating, and hypertension as coronary risk factors would generally be considered likely to have ACS but would be classified as low risk with a score of 13.

## 8. The 0-hour/1-hour Algorithm 

**Fig. 2.**
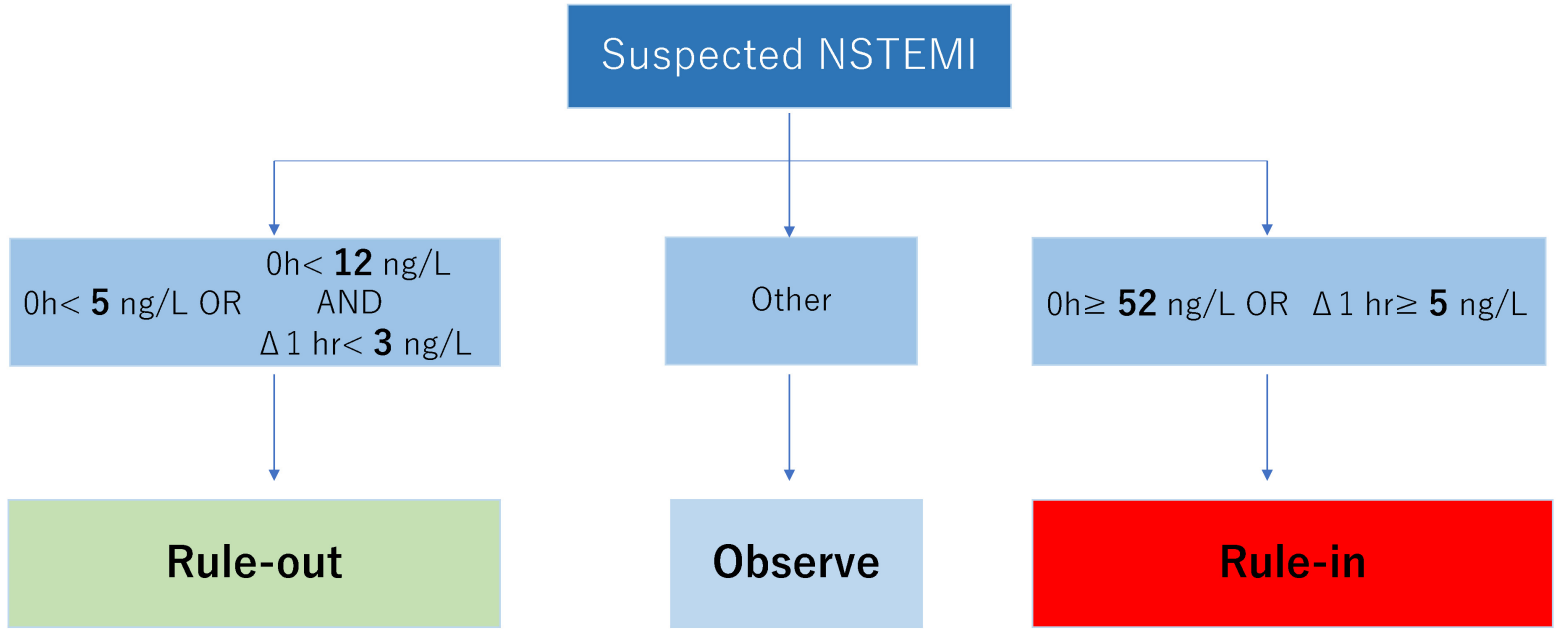
**The 0-hour/1-hour algorithm with high sensitivity cardiac troponin T**. The assay system to measure troponin must meet the conditions the International Federation of Clinical Chemistry recommends. NSTEMI, non-ST-elevation myocardial infarction.

Mueller *et al*. [27] at the University of Basel, Switzerland, proposed an algorithm to improve the efficiency of ACS diagnosis based on serial troponin measurements [28, 29]. Patients who present with chest pain as the primary complaint without evident electrocardiographic changes are assessed for high-sensitivity troponin at the time of arrival and one hour later, and the decision to send them home is based on their troponin levels. The advantages of this method are as follows (Fig. 2).

### 8.1 Serial Measurement

Based on Bayes’ theorem, if the first test is negative, the pre-diagnostic probability is lowered, and the second test is used to confirm (i.e., exclude) the first test, thus reducing the post-diagnostic probability. Initially, the second measurement depended on medical resources such as ED capacity, number of hospital beds, and staff. In extreme cases, the later it is, the more sufficient the increase in troponin levels and the fewer diagnostic errors. However, patients continued to be observed in the ED, leading to congestion. In 2012, a second measurement was recommended after three hours; however, a meta-analysis showed that it could be performed as early as one hour if a highly sensitive assay system was used [30].

### 8.2 Quantitative Representation

Diagnosis of ACS is often difficult because it involves qualitative expressions. This algorithm, which proposes a risk stratification based entirely on quantitative values, is a significant achievement which young residents can use.

## 9. Frequently Asked Questions about 0–1 h Algorithm

### 9.1 How do You Manage Observation Groups?

Approximately 20% of the patients in the observation group (25–30%) had Non-ST elevation (NSTE)-ACS [31, 32, 33, 34]. Current guidelines state that patients should be sent home after making appointments for coronary computed tomography angiography, exercise myocardial scintigraphy, or magnetic resonance imaging if there is no change in troponin levels after three hours. Lopez-Ayala [35] examined the optimal cutoff values using a sensitivity analysis of two cohort studies. The results showed that “high-sensitivity cardiac troponin T (hs-cTnT) <15 ng/L at three h and delta <4 ng/L” was the safest value for stratification, with a sensitivity of 93.3%, a negative predictive value of 94.7%, and an MI rate of 5%. However, even with this cutoff, approximately half of the patients were still stratified into the observation group (approximately 14.6% of the patients with ACS). With the recent development of machine learning, Doudesis *et al*. [36] created an interesting clinical decision support tool called the Collaboration for the Diagnosis and Evaluation of Acute Coronary Syndrome (CoDE-ACS). It was machine-trained with an XG Boost model using the High-STE-ACS cohort (10,038 patients (median age, 70 years; 48% female)) who were seen at one of 10 secondary or tertiary care hospitals in Scotland [36]. The external validation cohort included data from approximately 10,000 patients. CoDE-ACS was scored from 0–100, with an excellent diagnostic efficiency of 0.953 (0.947–0.958) for its AUC and a score of <3 to rule out AMI and ≥61 to suspect AMI. Only 3–60 points corresponded to the observation group of the 0–1 algorithm (12.8% included AMI); as a result, CoDE-ACS could be reduced to 13%.

### 9.2 Interpretation of Troponin Values in Patients with Heart Failure at Presentation

If a patient with heart failure show a troponin level above the 99th percentile, ischemic heart disease may be the underlying cause. However, in clinical practice, the first priority should be managing the heart failure itself. Coronary artery evaluation should only be considered if heart failure is not well controlled, rather than based on troponin values. The timing of coronary artery evaluation should be based on vital signs, including the patient’s respiratory status, rather than by troponin levels.

### 9.3 Can We Really Exclude Rule-out Groups?

Being classified in the exclusion group does not mean that coronary atherosclerosis is absent. In our cohort, 47% of patients were stratified into the rule-out group, and 6.7% underwent percutaneous coronary intervention (PCI) [6]. These patients had a HEART score of ≥4. Since around 90% of these patients underwent PCI within 30 days after index visit, they should be closely monitored in the outpatient setting for 30 days. After that, no patient experienced a cardiovascular event during one year.

### 9.4 On Admission Rule Out

Guidelines recommend excluding patients with baseline hs-cTnT levels below the detection limit (LoD) of 5 ng/L. This evidence is supported by meta-analyses [37] and two randomized trials using hs-cTnT [38, 39]. This may be a helpful tool to show patients that there is nothing to worry about, as hs-cTnT is also harmful in patients with a low pre diagnostic probability.

Clinicians should be aware of some of the analytical aspects of hs-cTn assays regarding the LoD and limit of quantitation. Since the guidelines define myocardial infarction as showing a rise and fall in troponin levels, it may be inappropriate to make judgments based solely on troponin values at the time of presentation.

### 9.5 No Information about the Characteristics of Patients

The 0–1 algorithm does not consider the patient’s background, age, or number of risk factors. Nilsson *et al*. [40] incorporated elements of the 0–1 algorithm into the HEART and EDAC scores, and both achieved an negative predictive value (NPV) >99.5% diagnostic performance for AMI at presentation. The HEART score combined with the 0–1 algorithm met the <1% miss rate guideline recommendation. However, neither method was superior to the other [41, 42]. Furthermore, the combined 0–1 algorithm with such a clinical score could exclude unstable angina.

Artificial intelligence technology, which has shown remarkable progress in recent years, is also beginning to benefit patients with chest pain. Neumann *et al*. [43] used machine learning to create a model for AMI diagnosis that incorporated 18 variables of patient information based on the BACC study (n = 2575, mixed groups of single and serial measurements), and StenoCario (n = 1688, mixed groups of single and serial measurements) was used for validation. Data from different countries were used to generalize the model. Diagnostic efficiency was excellent (AUC ranged from 0.92 to 0.98) when the prognostic predictors were AMI occurring within 30 days and all-cause mortality. In the future, a machine learning model incorporating ECG elements (multiple comparable ECGs recorded consecutively would be desirable) would allow safer and quicker risk stratification in patients with chest pain.

## 10. Conclusions

We reviewed the current status of how to approach patients with acute chest pain. This issue is long-standing, but has not been resolved. The reason why innovative methods for chest pain management have yet to be firmly established lies in the complexity and variability of chest pain etiology. Chest pain can originate from a wide range of causes, including cardiac, gastrointestinal, musculoskeletal, and psychological sources, making a one-size-fits-all approach challenging. Moreover, individual patient factors, such as comorbidities and varying presentations of symptoms, add layers of complexity to developing universally applicable and effective treatment protocols. The decision-making method that uses the technique with the highest empirical probability of resolution is called a heuristic. It is almost synonymous with the rules of thumb, quick thinking, and intuition. Decision-making is usually based on this process because it solves problems with less effort. The difference in the accuracy rates between experienced and young ED physicians can be attributed to the breadth of the heuristic repertoire. Heuristic bias, a negative aspect of heuristics, begins to appear when clinicians become slightly more familiar with them. Therefore, clinicians need to estimate the prior probability of diagnosis qualitatively from the medical interview and ECG within the limited time available in the ED and pay attention to increasing the post-test probability by quantitative evaluation using troponin to reduce the number of severe missed cases.

## Abbreviations

ACS, acute coronary syndrome; ECG, electrocardiogram; ED, emergency department; AMI, acute myocardial infarction; AUC, area under the curve; hs-cTn, high-sensitivity cardiac troponin; NSTE-ACS, non-ST-segment elevation acute coronary syndrome; CoDE-ACS, Collaboration for the Diagnosis and Evaluation of Acute Coronary Syndrome; LoD, limit of detection.
